# Association of TNF-α-308G/A, -238G/A, -863C/A, -1031T/C, -857C/T polymorphisms with periodontitis susceptibility: Evidence from a meta-analysis of 52 studies

**DOI:** 10.1097/MD.0000000000021851

**Published:** 2020-09-04

**Authors:** Lishuo Xu, Chenguang Liu, Youli Zheng, Yu Huang, Yang Zhong, Zhulan Zhao, Ning Ma, Zheng Zhang, Li Zhang

**Affiliations:** a Department of Periodontology, Jilin Stomatological Hospital, Hospital of Stomatology, Jilin University; b Department of Stomatology, Jilin Province People's Hospital, Changchun, Jilin; c Department of General Dentistry, Stomatological Hospital, Tianjin Medical University, Tianjin; d Department of Emergency, Jilin Stomatological Hospital, Hospital of Stomatology, Jilin University, Changchun, Jilin; e Department of Periodontology, Tianjin Stomatological Hospital and Tianjin Key Laboratory of Oral Function Reconstruction, Hospital of Stomatology, Nankai University, Tianjin, China.

**Keywords:** meta, periodontitis, polymorphisms, susceptibility, tumor necrosis factor-alpha

## Abstract

The association between tumor necrosis factor-alpha (TNF-α-308G/A, -238G/A, -863C/A, -1031T/C, and -857C/T) polymorphism and either chronic (CP) or aggressive (AgP) periodontitis susceptibility was conflicting. This meta-analysis aimed to quantitatively estimate the association.

A total of 52 studies involving 5519 patients and 7260 controls were identified through a search of multiple electronic databases. Odds ratios (ORs) and their 95% confidence intervals using allele, homozygous, heterozygous, dominant, and recessive genetic models were computed to assess the strength of the association.

The TNF-α-308G/A polymorphism was significantly associated with decreased risks of CP (GG vs AA: OR = 0.353, *P* < .001; GG+GA vs AA: OR = 0.480, *P* < .001) and AgP (G vs A: OR = 0.651, *P* < .001; GG vs AA: OR = 0.306, *P* < .001; GG+GA vs AA: OR = 0.384, *P* < .001) in Asians. There were no associations between TNF-α-238G/A, -863C/A, -1031T/C, -857C/T polymorphism and susceptibility to AgP. No associations were also found between CP susceptibility and TNF-α-238G/A, -857C/T polymorphism.

These findings supported that TNF-α-308G/A polymorphism might be the protective factors of CP and AgP in Asians, and TNF-α-238G/A, -863C/A, -1031T/C, -857C/T polymorphism is not linked to AgP susceptibility.

## Abstract 2

Supplemental Digital Content is available in the text

## 1 Introduction

Periodontal disease is a group of inflammatory disorders, primarily initiated by a chronic bacterial infection and related to the host response.^[1]^ It is one of the causes of tooth loss and can be correlated with systemic diseases, such as arthritis and diabetes.^[2]^ Majority of the population have experienced some levels of gingival inflammation worldwide, and 5% to 8% of the population suffering from severe forms of periodontitis.^[3]^ Periodontitis is divided into chronic periodontitis (CP) and aggressive periodontitis (AgP).^[4]^ There are many factors that cause periodontitis, including bacterial infection, genetic factors, and environmental factors. Common causes include stimulation of plaque, tartar, and smoking. Among them, plaque is considered to be the initiating factor for periodontitis.^[5,6]^ Plaques colonized in periodontal tissues can trigger the host's autoimmune and inflammatory responses, thereby affecting the progress and severity of the disease. In addition, some genetic factors can cause changes in the expression of encoded proteins and their levels, alter the host's immune and inflammatory response, and then affect the development of periodontitis.^[7]^

Numerous studies have shown that nearly half of the clinical differences in periodontal disease stem from genetic polymorphisms.^[8]^ Among these genes, tumor necrosis factor-alpha (TNF-α) has been considered as an important contributor to the pathogenesis of periodontal diseases. Bacterial pathogens in dental plaque can stimulate TNF-α secretion, cause osteoclast differentiation and bone resorption.^[9]^ TNF-α is produced by diverse kinds of cells including macrophages, neutrophils, keratinocytes, fibroblasts, T and B cells after stimulation.^[10]^ It is a potent proinflammatory cytokine and immune modulator with wide-ranging biological effects including protection from infection, surveillance against tumors, and stimulation of inflammatory responses.^[11–14]^ It also can trigger various immune and inflammatory process, including adhesion of polymorphonuclear leukocytes to endothelial cells,^[15]^ phagocyte a ctivation, and intercellular cell adhesion molecule-1 expression,^[16]^ as well as having roles in necrosis and apoptosis.

TNF-α gene is located in 6p21.3 and has several functional polymorphism sites. Single-nucleotide polymorphisms (SNP) within TNF-α have the potential to affect the function or regulation of TNF-α production.^[17]^ Several SNPs have been identified in its promoter. The TNF-α-308G/A (rs1800629), -238G/A (rs361525), and -863C/A(rs1800630) in the promoter region of the TNF-α gene are 3 common functional polymorphisms that have been demonstrated to be associated with the production level of the cytokine.^[18]^ The TNF-α-1031T/C (rs1799964), -857C/T(rs1799724) are newly discovered gene polymorphism that affect periodontitis susceptibility in recent years.^[19,20]^ A recent meta-analysis of TNF-α gene polymorphisms and susceptibility to periodontitis suggested that the TNF-α-308G/A and -863C/A AA genotypes contribute to susceptibility to CP, while the -308G/A polymorphism is connected with increased risk of AgP.^[18]^ During the past few years, the number of original studies linking TNF-α gene polymorphism to periodontitis has doubled. However, not all researchers confirmed the previous findings, and the controversy on this topic continues. In addition, no one has done a meta-analysis of the relationship between TNF-α-1031T/C, -857C/T gene polymorphism, and susceptibility to periodontitis. With accumulating evidence, we therefore aimed to examine the associations between TNF-α gene polymorphisms and periodontitis susceptibility by conducting an updated meta-analysis of original studies.

## 2 Materials and methods

Since all analyses were based on previously published studies, no ethical approval and patient consent were required.

### 2.1 Search strategy

Four electronic databases, namely, PubMed, Web of Science, Embase, and the Chinese National Knowledge Infrastructure databases, were searched in May 2019 by 2 independent reviewers (LX and ZZ). The following terms were used in this search: (Tumor Necrosis Factor-alpha OR TNF-alpha OR TNF-α OR rs1800629 OR rs361525 OR rs1800630 OR rs1799964 OR rs1799724) AND (polymorphism OR mutation OR variant) AND (periodontitis OR periodontal disease OR periodontal pocket OR alveolar bone loss OR attachment loss OR attachment level OR tooth mobility). Furthermore, references in related studies or reviews were also reviewed by hand searching to identify additional eligible studies.

### 2.2 Inclusion and exclusion criteria

The included studies must meet the following 5 criterias: All published case-control studies and cohort studies of TNF-α-308G/A, -238G/A, -863C/A, -1031T/C, and -857C/T gene polymorphisms related to susceptibility to periodontitis; For adults over 18 years of age, the clinical diagnosis was CP or AgP, the correct diagnostic criteria and methods of CP and AgP were clearly mentioned, and the control group was a normal healthy population; The original literature had the allele and genotype distribution data that was detailed or could calculate through the data provided in the article, and could calculate odds ratio (OR) and 95% confidence interval (95%CI); Included studies were research reports published in English or Chinese with full text.

The exclusion criteria were as follows: Nonmedical case-control literature; Duplicate publication; Data was incomplete, genotype frequency couldn’t be calculated, and experimental results were unclear; There were obvious design flaws, and the quality of the literature was poor.

### 2.3 Quality assessment

The quality of each study was evaluated independently by 2 reviewers (LX and ZZ) according to the criteria described by Nibali.^[21]^ The categories in scoring system used for assessing study quality were as follows: selection, comparability, exposure, study methodology/design, and genetic analyses. The details of each methodological item were listed in Supplementary Table S1. Scores ranged from 0 (lowest) to 20 (highest), and studies with scores ≥10 were classified as high-quality studies, whereas studies with scores < 10 were classified as low-quality studies.

### 2.4 Data extraction

Two investigators (LX and ZZ) individually checked the titles and abstracts, and then selected the relevant full-text articles, according to the inclusion criteria. Any disagreement was resolved via discussion or adjudicated by a third author. The following information was collected from each study: first author, publication year, country/region, the number of cases and controls, the racial descent-Caucasian, Asian and Mixed (If the authors did not clearly report the ethnic information or we could not separate them according to genotypes distribution, the ethnicity was classified as “Mixed”), clinical types of periodontitis, genotyping method, and genotype/allele distribution in both cases and controls, and evidence of Hardy-Weinberg equilibrium (HWE). A *P* value less than .05 of HWE was considered significant.

### 2.5 Statistical analysis

Statistical analysis was performed by using Stata software (Version 12.0, Stata Corp, College Station, TX). The associations of the TNF-α gene polymorphisms and susceptibility to periodontitis were estimated by calculating the pooled ORs and 95% CIs. This study used Bonferroni correction method, and the adjusted significance threshold was set at 0.01 (0.05/5) for single polymorphism since 5 polymorphisms were analyzed. Heterogeneity was tested by the Q test and I^2^ statistics. If the result of the Q test was *P* value > .05 and I^2^ < 50%, indicating the absence of heterogeneity, then a fixed-effects model was used to estimate the summary ORs; otherwise, the random-effects model was used. The rank correlation method of Begg^[22]^ and Egger^[23]^ linear regression method were used to evaluate potential publication bias. *P* value < .05 was used as an indication for the presence of potential publication bias.

## 3 Results

### 3.1 Literature search

A total of 303 published articles were identified from 4 databases, and 3 articles were found by hand research. When we removed the duplicates, 156 articles were selected. After reading the title and abstract, 87 of these articles were excluded, leaving 69 studies for full publication review. Finally, 52 studies containing a total of 5519 patients and 7260 controls were included in this meta-analysis (Supplementary Figure S1).

### 3.2 Study characteristics

The main characteristics of the included studies were summarized in Supplementary Table S2. Among these studies, 45 studies investigated the -308G/A polymorphism, 10 studies investigated the -238G/A polymorphism, 8 studies investigated the -863C/A polymorphism, 5 studies investigated the -1031T/C polymorphism, 7 studies investigated the -857C/T polymorphism. Twenty-three studies were performed in Asians, 21 studies were performed in Caucasians, and 8 studies were performed in Mixed. In terms of the type of periodontitis, 31 studies focused on CP, 10 studies focused on AgP and 11 studies focused on both CP and AgP. In the 52 included studies, 41 studies were in accordance with HWE for the genotype distribution, whereas 7 studies represented a significant departure from HWE.

### 3.3 TNF-α-308G/A(rs1800629) polymorphism

Significant association of the -308G/A polymorphism with protective factors for CP was only detected in Asians under homozygous model and dominant model (GG vs AA: OR=0.353, 95% CI: 0.241–0.515, *P* < .001; GG+GA vs AA: OR = 0.480, 95% CI: 0.338–0.684, *P* < .001) (Table 1 and Supplementary Figure S2). Significant heterogeneity was seen in Asians under the allele and recessive models. To explore potential source of heterogeneity across the studies, we performed sensitivity analyses. The results indicated that 3 studies^[24–26]^ might be the major source of the heterogeneity in Asians. When we excluded these studies and repeated the analysis, heterogeneity was no longer present, and significant association was also found in Asians under the allele and recessive models (G vs A: OR = 0.587, 95% CI: 0.445–0.774, *P* < .001; GG vs GA+AA: OR = 0.466, 95% CI: 0.361–0.602, *P* < .001) (Supplementary Figure S4-S5). After exclusion of the studies that deviated from HWE, significantly correlations were found in Asians in 4 genetic models tested (G vs A: OR = 0.491, 95% CI: 0.395–0.609, *P* < .001; GG vs AA: OR = 0.292, 95% CI: 0.145–0.590, *P* = .001; GG vs GA+AA: OR = 0.468, 95% CI: 0.367–0.596, *P* < .001; GG+GA vs AA: OR = 0.337, 95% CI: 0.164–0.693, *P* = .003) (Table 1).

**Table 1:**
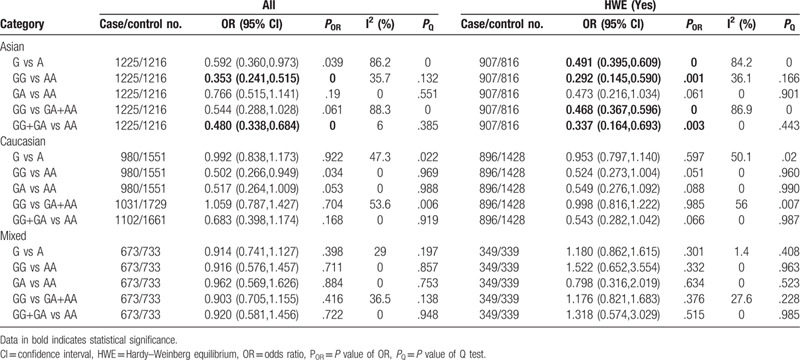
Meta-analysis of the association between TNF-α-308G/A(rs1800629) polymorphism and chronic periodontitis.

The results showed significant associations of the -308G/A polymorphism with AgP in Asians under the allele (G vs A: OR = 0.651, 95% CI: 0.526–0.806, *P* < .001), homozygous (GG vs AA: OR = 0.306, 95% CI: 0.169–0.551, *P* < .001), recessive (GG vs GA+AA: OR = 0.677, 95% CI: 0.523–0.876, *P* = .003), and dominant (GG+GA vs AA: OR = 0.384, 95% CI: 0.227–0.650, *P* < .001) models, but not in Caucasians and Mixed (Table 2 and Supplementary Figure S6, S7, S8 & S9). And there was no evidence of statistical heterogeneity in all populations. After exclusion of the studies that deviated from HWE, significantly correlations were found in Asians in 4 genetic models (G vs A: OR = 0.717, 95% CI: 0.565–0.909, *P* = .006; GG vs AA: OR = 0.317, 95% CI: 0.143–0.699, *P* = .004; GA vs AA: OR = 0.315, 95% CI: 0.138–0.716, *P* = .006; GG+GA vs AA: OR = 0.318, 95% CI: 0.146–0.693, *P* = .004) and in Caucasians (G vs A: OR = 0.606, 95% CI: 0.417–0.880, *P* = .009) (Table 2).

**Table 2:**
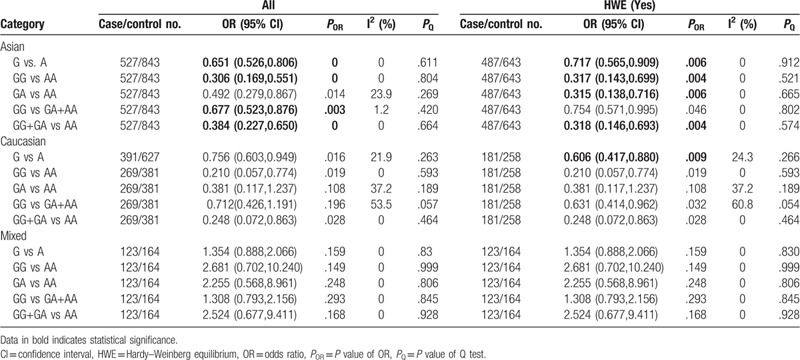
Meta-analysis of the association between TNF-α-308G/A (rs1800629) polymorphism and aggressive periodontitis.

### 3.4 TNF-α-238G/A(rs361525) polymorphism

The results of the associations between TNF-α-238G/A polymorphism and CP susceptibility were that no model was statistically significant in Asians and Caucasians. And there were greater heterogeneity in the 2 races, when we performed sensitivity analysis, the exclusion of the studies^[27,28]^ with heterogeneity did not change the results significantly. After excluding the HWE violation study, the results were still not statistically significant (Table 3). The relations of TNF-α-238G/A polymorphism and AgP susceptibility were also no statistical correlation (Table 4).

**Table 3:**
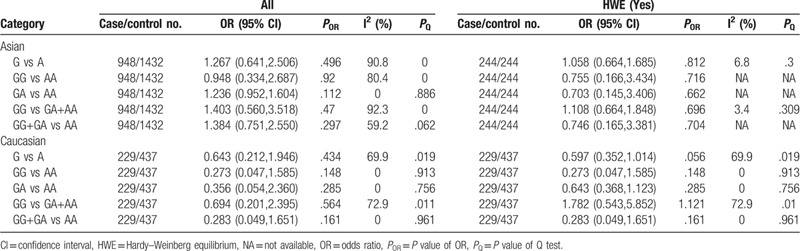
Meta-analysis of the association between TNF-α-238G/A (rs361525) polymorphism and chronic periodontitis.

**Table 4:**
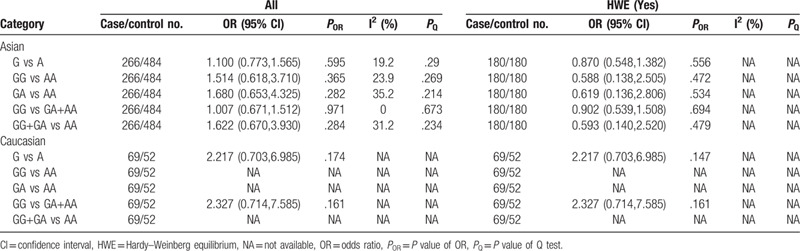
Meta-analysis of the association between TNF-α-238G/A (rs361525) polymorphism and aggressive periodontitis.

### 3.5 TNF-α-863C/A(rs1800630) polymorphism

The results of the associations between TNF-α-863C/A polymorphism and CP susceptibility were that no model was statistically significant. And the exclusion of the studies^[29]^ with heterogeneity did not change the results significantly. And after exclusion of the studies that deviated from HWE, significantly correlations were found in Asians in 2 genetic models tested (C vs A: OR = 0.661, 95% CI: 0.515–0.849, *P* = .001; CC vs CA+AA: OR = 0.654, 95% CI: 0.488–0.877, *P* = .005) (Table 5). The relations of TNF-α-863C/A polymorphism and AgP susceptibility were also no statistical correlation (Table 6).

**Table 5:**
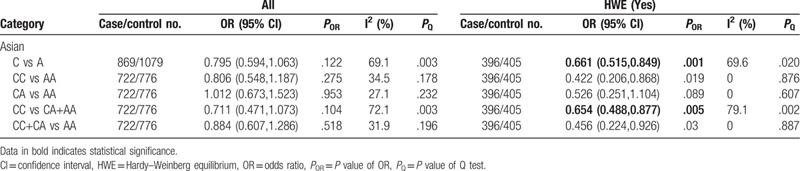
Meta-analysis of the association between TNF-α-863C/A (rs1800630) polymorphism and chronic periodontitis.

**Table 6:**
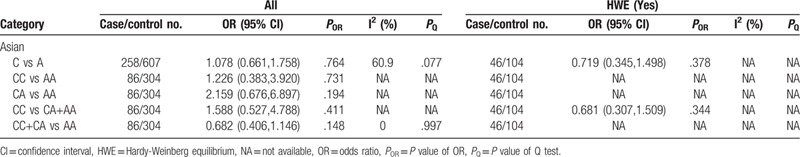
Meta-analysis of the association between TNF-α-863C/A (rs1800630) polymorphism and aggressive periodontitis.

### 3.6 TNF-α-1031T/C(rs1799964) polymorphism

In Asians, significant association was found between the TNF-α-1031T/C polymorphism and CP susceptibility under the homozygous model (TT vs CC: OR = 0.557, 95% CI: 0.388–0.798, *P* = .001) (Table 7 and Supplementary Figure S10). And the results of the relation of TNF-α-1031T/C polymorphism and AgP susceptibility were that no model was statistically significant (Table 8). When we performed sensitivity analysis, the exclusion of the studies^[20,26]^ with heterogeneity did not change the results significantly. In addition, when stratified by HWE studies, all findings were negative.

**Table 7:**
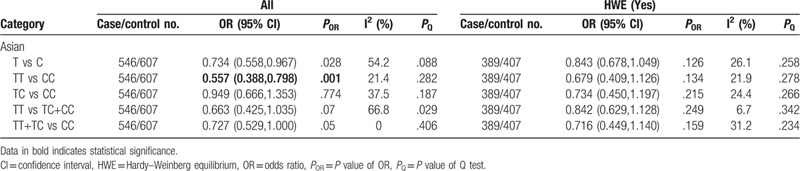
Meta-analysis of the association between TNF-α-1031T/C (rs1799964) polymorphism and chronic periodontitis.

**Table 8:**
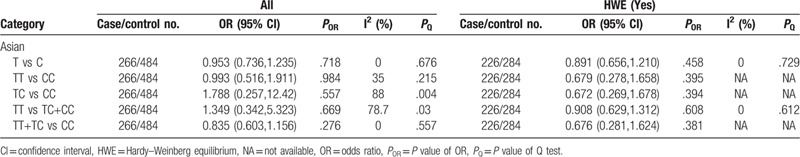
Meta-analysis of the association between TNF-α-1031T/C (rs1799964) polymorphism and aggressive periodontitis.

### 3.7 TNF-α-857C/T(rs1799724) polymorphism

Five studies^[20,26,30–32]^ investigated TNF-α-857C/T polymorphism and its associations with CP. Another 5 studies^[19,20,26,30,33]^ investigated -857C/T polymorphism and its associations with Agp. Heterogeneity tests showed that the associations of -857C/T gene polymorphisms with CP and AgP in Asians were derived from stable results. So we used fixed-effects models and no model was statistically significant (Tables 9 and 10). When stratified by HWE studies, all findings were negative.

**Table 9:**
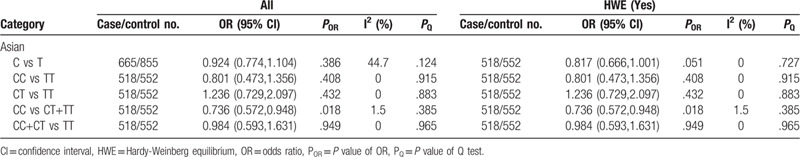
Meta-analysis of the association between TNF-α-857C/T (rs1799724) polymorphism and chronic periodontitis.

**Table 10:**
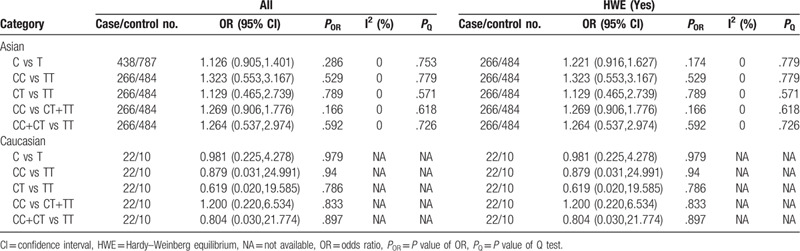
Meta-analysis of the association between TNF-α-857C/T (rs1799724) polymorphism and aggressive periodontitis.

### 3.8 Publication bias

Begg funnel plot and Egger test were performed to assess the publication bias of included studies. As shown in Supplementary Figure S11, the shapes of the funnel plots did not reveal any indication of publication bias. Besides, Egger test was used to provide statistical evidence of funnel plot symmetry, and the results did not show any evidence of publication bias. All *P* values from the Egger test were > .05 (data not shown).

## 4 Discussion

TNF-α, also known as cachexia or TNFSF1A, is a prototype ligand of the TNF superfamily. It is a pleiotropic molecule that plays an important role in inflammation, immune system development, apoptosis, and lipid metabolism.^[34]^ It is also a pro-inflammatory cytokine with immunoregulatory function. It has extensive biological effects on leukocytes, vascular endothelial cells, and other cells in connective tissue. TNF-α can lead to the destruction of connective tissue and enhance the formation and activity of osteoclasts, finally limiting the repair of periodontal tissue.^[35]^

To date, numerous studies evaluated the association between TNF-α gene polymorphisms and periodontitis susceptibility have been published, but the results were inconsistent. A meta-analysis first published by Nikolopoulos GK in 2008^[36]^ (involving15 studies) showed that there was no association of the TNF-α-308G/A gene polymorphism with periodontitis. Other meta-analysis published in 2013^[37]^ (involving 17 studies for -308G/A,3 studies for -238G/A) found that TNF-α-308G/A A allele was associated with periodontitis in Brazilian, Asian, and Turkish populations, no association between the TNF-α-238G/A gene polymorphism and periodontitis. The recent meta-analysis published in 2014^[18]^ (31 studies for -308G/A, 7 studies for -238G/A, and 6 studies for -863C/A) concluded that the TNF-α-308G/A and -863C/A AA genotypes contributed to susceptibility to CP, while the -308G/A polymorphism was associated with increased risk of AgP. No considerable association was identified between TNF-α-238G/A polymorphism and CP. Recently, many new studies subsequent to these meta-analyses have emerged, but a consensus has not been reached. Therefore, there is a need to carry out this updated meta-analysis to summarize the latest relationship between TNF-α gene polymorphism and periodontitis susceptibility.

To our knowledge, this meta-analysis is so far the largest that had evaluated TNF-α polymorphisms with periodontitis susceptibility. Thus, the results of our meta-analysis were more reliable than those of previous studies. In this meta-analysis, we collected all the related studies up to date and re-evaluated the association between -308G/A polymorphism and periodontitis. We found that the genotype GG and the GG + GA in the dominant model (GG+GA vs AA) were the protective factors of CP. After conducting a sensitivity analysis to remove the study of greater heterogeneity and reanalysis, we found that the allele G was also the protective factor of CP in Asians. When limited to HWE studies, associations were also found in 4 genetic models in Asians.

In the AgP study, it was also found that there was a statistical correlation between the TNF-α-308G/A gene polymorphism and the susceptibility to AgP in the Asians. This indicated that the allele G, GG genotype, and the GG+GA in dominant model (GG+GA vs AA) were the protective factors of AgP. When limited to HWE studies, associations were also found in 4 genetic models in Asians. For both CP and AgP, we observed the effects of TNF-α gene polymorphisms and susceptibility to periodontitis for Asians were stronger than Caucasians and Mixed. Therefore, we speculated that there may be ethnic differences in the correlation between the TNF-α-308G/A gene polymorphism and the susceptibility to periodontitis.

For the TNF-α-238G/A gene polymorphism, we saw no model was statistically significant in the original study and HWE study in CP and AgP. This indicated that -238G/A gene polymorphism had no connection with periodontitis susceptibility. This result was the same as the meta-analysis which in 2014.^[18]^ Shungin et al^[38]^ and Masumoto et al^[39]^ also recently reported no association between the -238G/A gene polymorphism and CP susceptibility as reported in the genome-wide association study. In the future, we still need a lot of time and data to explore the relationship between -238G/A gene polymorphism and susceptibility to periodontitis.

There were many studies on the correlation between -863C/A gene polymorphism and disease. In 1999, Skoog et al^[40]^ found that -863C/A was associated with the disease severity. A SNP at position -863 was involved in NF-κB binding affecting the transcriptional regulation. TNF-863A allele lessened the NF-κB p50/p50 binding that directed the enhanced TNF production in human monocytes.^[41]^ In 2003, Soga's study demonstrated that −863C/A A allele was a risk factor for CP.^[32]^ These meaned -863C/A gene polymorphism may have impact on disease. However, we observed no relationship between the TNF-α-863C/A gene polymorphism and susceptibility to CP and AgP in this meta-analysis. The results were completely opposite to the previous analysis. It may be because we included much more studies than the previous ones. As new researches accumulated, the correlation changes from significant to nonsignificant. Thus, more researches are still required to verify the association between -863C/A gene polymorphism and periodontitis.

Our study was the first meta-analysis documented an association between TNF-α-1031T/C gene polymorphisms and periodontitis susceptibility. Our data showed that TT genotype may be an important protective factor for CP patients in Asians. A recent study showed^[42]^ in -1031T/C gene polymorphisms, homogeneous risk allele -1031C genotype had remarkably higher TNF expression than homogeneous reference allele -1031T genotype, this finding was confirmed in our meta-analysis. In addition, some studies have shown that TNF-α-863C/A and TNF-α-1031T/C could stimulate expression and protein secretion of TNF RNA.^[43–46]^ Therefore, we speculated that TNF-α-1031T/C may also control the progression of periodontitis by increasing RNA expression and cytokine levels.

To our knowledge, the present study was also the first meta-analysis which assessed the possible influence of TNF-α-857C/T gene polymorphism on susceptibility to periodontitis. Based on analysis results, our study suggested that -857C/T gene polymorphisms were not related to periodontitis. Due to the small sample size and single ethnicity included, this result was unstable. We need to conduct high-quality research in large populations to get more accurate results.

Several limitations of this meta-analysis should be noticed. First, studies included in our meta-analysis were mainly English and Chinese published articles, which may have caused a selection bias. Second, we did not think out gene–gene and gene–environmental interactions in the case-control study. Third, the number of included studies in the meta-analysis was still small, we could not perform a more precise analysis based on adjusted information. Fourth, the authors observed the presence of elevated heterogeneity in some comparisons herein presented. Beyond the statistical heterogeneity, there was also the clinical heterogeneity. Periodontitis and its diagnosis were characterized by variations since severity and forms of disease until diagnosis, what may be a potential bias in studies. Therefore, the results should be interpreted with caution.

## 5 Conclusions

In spite of the limitations mentioned above, the results of this meta-analysis suggested that TNF-α-308G/A polymorphism might be the protective factors of CP and AgP in Asians, and TNF-α-238G/A, -863C/A, -1031T/C, -857C/T polymorphism is not linked to AgP susceptibility. However, given the limitations of this meta-analysis, we cannot obtain a conclusive result. In future, additional well-designed studies are necessary to clarify these relationships and thus reinforce our findings.

## Author contributions

**Data curation:** Lishuo Xu.

**Methodology:** Lishuo Xu, Youli Zheng.

**Project administration:** Chenguang Liu.

**Writing – original draft:** Lishuo Xu, Chenguang Liu.

**Writing – review & editing:** Youli Zheng.

## Supplementary Material

Supplemental Digital Content

## Supplementary Material

Supplemental Digital Content

## Supplementary Material

Supplemental Digital Content

## Supplementary Material

Supplemental Digital Content

## Supplementary Material

Supplemental Digital Content

## Supplementary Material

Supplemental Digital Content

## Supplementary Material

Supplemental Digital Content

## Supplementary Material

Supplemental Digital Content

## Supplementary Material

Supplemental Digital Content

## Supplementary Material

Supplemental Digital Content

## Supplementary Material

Supplemental Digital Content

## Supplementary Material

Supplemental Digital Content

## Supplementary Material

Supplemental Digital Content
