# Supplementary material for: Association of TNF-α-308G/A, -238G/A, -863C/A, -1031T/C, -857C/T polymorphisms with periodontitis susceptibility: Evidence from a meta-analysis of 52 studies
Source: Medicine (Baltimore). 2020 Sep 4;99(36):e21851. doi: 10.1097/MD.0000000000021851 (PMC7478382; doi:10.1097/MD.0000000000021851)
Supplement: Supplemental Digital Content [file medi-99-e21851-s010.docx]

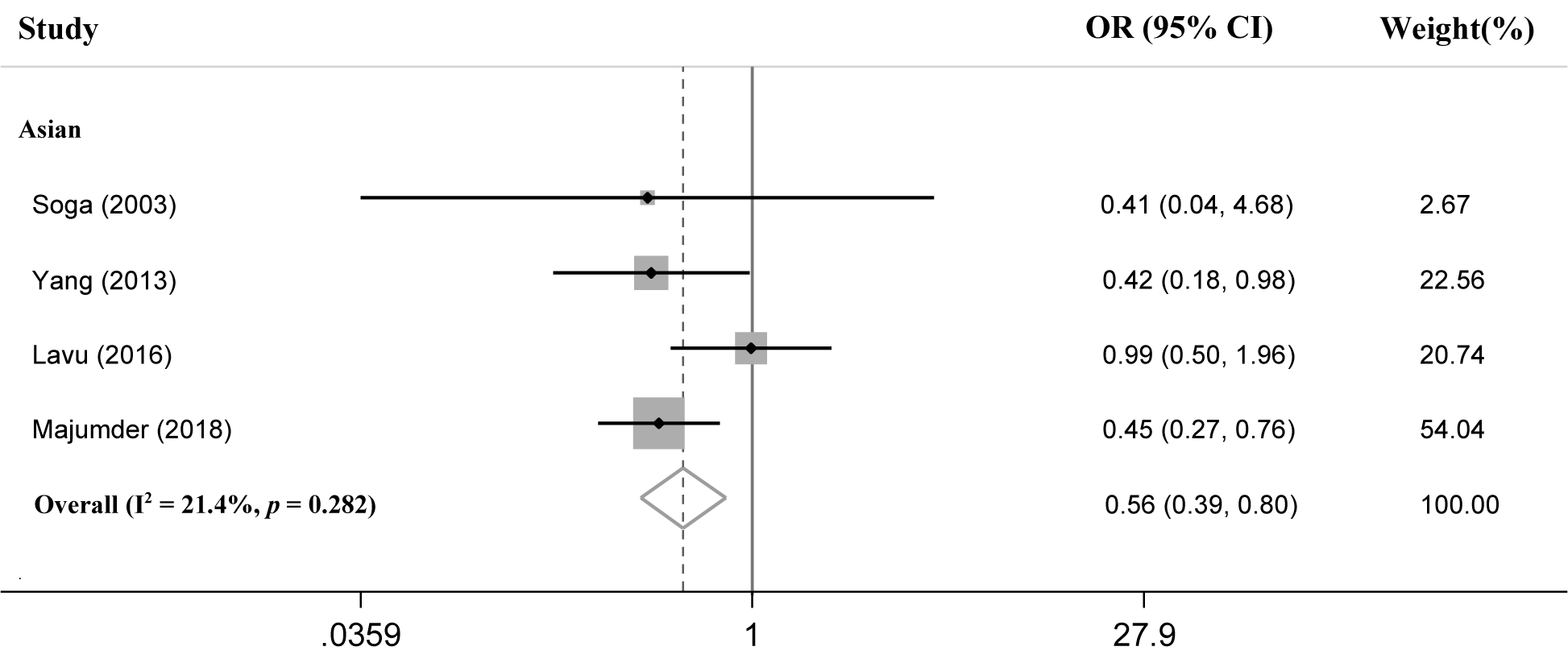


**Figure S10** Meta-analysis of TNF-α-1031T/C polymorphism with chronic periodontitis under homozygous model (TT vs.CC).
