# Supplementary material for: Association of TNF-α-308G/A, -238G/A, -863C/A, -1031T/C, -857C/T polymorphisms with periodontitis susceptibility: Evidence from a meta-analysis of 52 studies
Source: Medicine (Baltimore). 2020 Sep 4;99(36):e21851. doi: 10.1097/MD.0000000000021851 (PMC7478382; doi:10.1097/MD.0000000000021851)
Supplement: Supplemental Digital Content [file medi-99-e21851-s012.docx]

**Table S1** Assessing the quality of included studies.

| **Author (year)** | **Selection** | **Comparability** | **Exposure** | **Study methodology/Design** | **Genetic analyses** | **Score** |
| --- | --- | --- | --- | --- | --- | --- |
| Galbraith (1998) | ●○●● | ○ | ●●○ | ○●●● | ●●●●●○○● | 14 |
| Galbraith (1999) | ●○●● | ○ | ●●○ | ○●●● | ●●●●●○○● | 14 |
| Endo (2001) | ●○●● | ○ | ●●○ | ○●○○ | ●●●●●○○● | 12 |
| Shapira (2001) | ●○○○ | ● | ●●○ | ○○○○ | ○○●○●●●○ | 8 |
| Craandijk (2002) | ●○○● | ● | ●●○ | ●●○● | ●●●●●○○● | 14 |
| Qian (2002) | ●○●● | ● | ●●○ | ○●○○ | ●●●○●○○● | 12 |
| Fassmann (2003) | ●○●● | ● | ●●○ | ○●●● | ●●●●●○○● | 15 |
| Soga (2003) | ●○●● | ● | ●●○ | ○●○● | ●●●●●○○● | 14 |
| Folwaczny (2004) | ●○●● | ○ | ●●○ | ○●○○ | ●●●●●○○● | 12 |
| Nan (2004) | ●○●● | ● | ●●○ | ○●○○ | ●●●●●○○● | 13 |
| Brett (2005) | ●○●● | ○ | ●●○ | ●●○● | ●●●○●○○● | 13 |
| Donati (2005) | ●○●● | ● | ●●○ | ○●○○ | ●●●●●○○● | 13 |
| Zhong (2005) | ●○●● | ● | ●●○ | ○●○○ | ●●●●●○○● | 13 |
| Pang (2005) | ●○●● | ● | ●●○ | ○●○● | ●●●●●●●● | 16 |
| Bable (2006) | ●○●● | ● | ●●○ | ○●○● | ●●●○●○○○ | 12 |
| Sakellari (2006) | ●○●● | ● | ●●○ | ○●○○ | ●●●●●○○● | 13 |
| de Sa´(2007) | ●○●● | ● | ●●○ | ○●○○ | ●●●●●●○● | 14 |
| de Freitas (2007) | ●○●● | ● | ●●○ | ●●○○ | ●●●●●○○● | 14 |
| Zhu (2007) | ●○●● | ● | ●●○ | ○●○● | ●●●●●○○● | 14 |
| Tervonen (2007) | ●○●● | ● | ●●○ | ○●○○ | ●●●○●○○○ | 11 |
| Guzeldemir (2008) | ●○●● | ● | ●●○ | ○●○● | ●●●●●○○● | 14 |
| Menezes (2008) | ●○○● | ○ | ●●○ | ○●○● | ●●●●●○○● | 12 |
| Schulz (2008) | ●○●● | ● | ●●○ | ●●●● | ●●●●●○○● | 16 |
| Wang (2008) | ●○●● | ● | ●●○ | ○●○○ | ●●○●●○○○ | 11 |
| Kobayashi (2009) | ●○●● | ● | ●●○ | ●●●● | ●●●●●●●● | 18 |
| Kobayashi (2009) | ●○●● | ● | ●●○ | ●●○● | ●●○○●●●○ | 14 |
| Moreira (2009) | ●○○● | ● | ●●○ | ○●○○ | ●●●●●○○● | 12 |
| Li (2009) | ●○●● | ○ | ●●○ | ○●○○ | ●●●●●○○● | 12 |
| Sun (2009) | ●○●● | ● | ●●○ | ○●○● | ●●●●●●●● | 16 |
| Trombone (2009) | ●○●● | ● | ●●○ | ○●○● | ●●●○●○○● | 13 |
| Costa (2010) | ●○○● | ● | ●●○ | ○●○○ | ●●●●●○○● | 12 |
| Erciyas (2010) | ●○●● | ● | ●●○ | ○●○○ | ●●●●●○○● | 13 |
| Ricci (2011) | ●○●● | ○ | ●●○ | ●●○● | ●●●●●○○● | 14 |
| Liu (2011) | ●○●● | ○ | ●●○ | ○●○○ | ●●●●●●●● | 14 |
| Zhang (2011) | ●○●● | ● | ●●○ | ○●○● | ●●●●●○○● | 14 |
| Ma (2011) | ●○●● | ● | ●●○ | ○●○● | ●●●○●○○● | 13 |
| Scapoli (2011) | ●○●● | ● | ●●○ | ○●●● | ●●○●●●●○ | 15 |
| Loo (2012) | ●○●● | ○ | ●●○ | ●●○● | ●●●○●○○● | 13 |
| Garlet (2012) | ●○●● | ● | ●●○ | ●●●● | ●●●○●●●● | 17 |
| Ianni (2013) | ●○●● | ○ | ●●○ | ○●●● | ●●●○○●○● | 13 |
| Yang（2013） | ●○●● | ● | ●●○ | ○●○● | ●●●●●○○● | 14 |
| Ebadian (2013) | ●○●● | ● | ●●○ | ○●○○ | ●●●●●○○● | 13 |
| Sharma（2014） | ●○●● | ● | ●●○ | ○●●○ | ●●●●●●○● | 15 |
| Schulz（2014） | ●○○○ | ○ | ●●○ | ○●○● | ○○●○○●●○ | 8 |
| Özer Yücel（2015） | ●○●● | ○ | ●●○ | ○●○○ | ●●●○●●○● | 12 |
| Dosseva-panova（2015） | ●○○● | ○ | ●●○ | ○●○○ | ●●●○●●○● | 11 |
| Ho（2015） | ●○●● | ○ | ●●○ | ○●○● | ●●●●●●○● | 14 |
| Barnea（2015） | ●○●● | ○ | ●●○ | ○●○○ | ●●●○○●○● | 11 |
| Grigorovich（2015） | ●○○● | ○ | ●●○ | ○●○○ | ●●●○○●○● | 10 |
| Lavu（2016） | ●○●● | ○ | ●●○ | ○●●● | ●●●●●●○● | 15 |
| Dominguez（2017） | ●○●● | ○ | ●●○ | ○●○● | ●●●●○●○● | 13 |
| Majumder（2018） | ●○●● | ○ | ●●○ | ○●○● | ●●●●●●○● | 14 |
| **Category** | **Item** | | | **Positive answer (score 1)** | | |
| Selection | Adequate case definition | | | Full mouth probing and accepted independently defined diagnosis adopted (e.g. EFP diagnoses) (or validated diagnosis by scoring bone loss in full mouth radiographs) | | |
|  | Representativeness of cases | | | Consecutive or obviously representative cases selected (no evidence of selection bias) | | |
|  | Selection of controls | | | Community/hospital controls geographically and ethnically matched to the cases | | |
|  | Definition of controls | | | Absence of disease assessed (full mouth probing done and accepted independently defined diagnosis of health adopted) | | |
| Comparability | Confounders | | | Cases and controls balanced for smoking, socio-economic status, BMI (or confounders adjusted for) | | |
| Exposure | Ascertainment of exposure | | | Secure record | | |
|  | Same method of ascertainment for cases and controls | | | Yes | | |
|  | Non-response rate | | | Same for cases and controls | | |
| Study methodology/ design | Power calculation | | | Performed a priori | | |
|  | Statistics | | | Well-described tests of significance for primary outcome | | |
|  | Corrected statistics | | | Correction for false positive (type I) error | | |
|  | Odds ratios and confidence intervals | | | Provided | | |
| Genetic analyses | Success rate of DNA extraction | | | Reported good rates | | |
|  | Success rate of genetic assessment | | | Reported good rates | | |
|  | Genotype counts | | | Provided in table or text | | |
|  | Hardy–Weinberg equilibrium | | | Satisfied | | |
|  | Primer sequence | | | Provided or referenced | | |
|  | Reproducibility | | | Described genotyping method to allow replication, validated genotyping accuracy | | |
|  | Genotyping blind to case–control status | | | Yes | | |
|  | Proposed inheritance model | | | Inheritance model-free approach (not exclusively dominant or recessive pattern proposed) | | |

●Yes ○No
