# Supplementary material for: Association of TNF-α-308G/A, -238G/A, -863C/A, -1031T/C, -857C/T polymorphisms with periodontitis susceptibility: Evidence from a meta-analysis of 52 studies
Source: Medicine (Baltimore). 2020 Sep 4;99(36):e21851. doi: 10.1097/MD.0000000000021851 (PMC7478382; doi:10.1097/MD.0000000000021851)
Supplement: Supplemental Digital Content [file medi-99-e21851-s013.docx]

**Table S2** Characteristics of studies included in the meta-analysis.

| **First author** | **Year** | **Country/region** | **Ethnicity** | **Case/control**  **Number** | **Genotyping method** | **Periodontitis type** | **Polymorphisms** | **HWE** | | | | |
| --- | --- | --- | --- | --- | --- | --- | --- | --- | --- | --- | --- | --- |
|  |  |  |  |  |  |  |  | **-308** | **-238** | **-863** | **-1031** | **-857** |
| Galbraith ^[40]^ | 1998 | USA | Caucasian | 32/32 | PCR-RFLP | CP | -308,-238 | YES | YES |  |  |  |
| Galbraith ^[39]^ | 1999 | USA | Caucasian | 19/45 | PCR | CP | -308 | YES |  |  |  |  |
| Endo ^[35]^ | 2001 | Japan | Asian | 46/104 | PCR-SSOP | AgP | -308,-238,-863,-1031,-857 | YES | NO | YES | YES | YES |
| Shapira ^[58]^ | 2001 | Israel | Asian | 16/27 | PCR | AgP | -308 | YES |  |  |  |  |
| Craandijk ^[28]^ | 2002 | Netherland | Caucasian | 90/264 | PCR-SSOP | CP | -308,-238 | YES | YES |  |  |  |
| Qian ^[53]^ | 2002 | China | Asian | 65/96 | PCR-RFLP | CP | -308 | NO |  |  |  |  |
| Fassmann ^[37]^ | 2003 | Czech Republic | Caucasian | 132/114 | PCR-RFLP | CP | -308 | YES |  |  |  |  |
| Soga ^[60]^ | 2003 | Japan | Asian | 64/64 | PCR-RFLP | CP | -308,-238,-863,-1031,-857 | YES | YES | YES | YES | YES |
| Folwaczny ^[38]^ | 2004 | Germany | Caucasian | 81/80 | PCR-RFLP | CP | -308 | YES |  |  |  |  |
| Nan ^[50]^ | 2004 | China | Asian | 45/85 | PCR-RFLP | CP | -308 | YES |  |  |  |  |
| Brett ^[25]^ | 2005 | UK | Caucasian | 55/97 | PCR-RFLP | CP | -308 | NO |  |  |  |  |
|  |  |  |  | 50/97 |  | AgP |  | NO |  |  |  |  |
| Donati ^[32]^ | 2005 | Sweden | Caucasian | 60/39 | PCR-RFLP | CP | -308 | YES |  |  |  |  |
| Zhong ^[66]^ | 2005 | China | Asian | 133/92 | PCR-SSP | CP | -308 | YES |  |  |  |  |
| Pang ^[52]^ | 2005 | China | Asian | 166/80 | PCR-RFLP | CP | -308 | YES |  |  |  |  |
| Bable ^[24]^ | 2006 | Germany | Caucasian | 122/110 | PCR-SSP | CP | -308 |  |  |  |  |  |
| Sakellari ^[55]^ | 2006 | Greece | Caucasian | 56/90 | PCR-RFLP | CP | -308 | YES |  |  |  |  |
|  |  |  |  | 46/90 |  | AgP |  | YES |  |  |  |  |
| de Sa´^[30]^ | 2007 | Brazil | Mixed | 45/53 | PCR | CP | -308 | YES |  |  |  |  |
| de Freitas ^[29]^ | 2007 | Brazil | Mixed | 30/70 | PCR-RFLP | AgP | -308 | YES |  |  |  |  |
| Zhu ^[67]^ | 2007 | China | Asian | 64/78 | PCR-RFLP | AgP | -308 | YES |  |  |  |  |
| Tervonen ^[62]^ | 2007 | Finland | Caucasian | 51/178 | PCR-RFLP | CP | -308 |  |  |  |  |  |
| Guzeldemir ^[43]^ | 2008 | Turkey | Caucasian | 31/31 | PCR-RFLP | AgP | -308 | YES |  |  |  |  |
| Menezes ^[48]^ | 2008 | Brazil | Mixed | 74/51 | PCR-RFLP | CP | -308 | YES |  |  |  |  |
|  |  |  |  | 38/51 |  | AgP |  | YES |  |  |  |  |
| Schulz ^[56]^ | 2008 | Germany | Caucasian | 54/52 | PCR-SSP | CP | -308,-238 | YES | YES |  |  |  |
|  |  |  |  | 69/52 |  | AgP |  | YES | YES |  |  |  |
| Wang ^[64]^ | 2008 | China | Asian | 33/33 | PCR-RFLP | AgP | -308 | YES |  |  |  |  |
| Kobayashi ^[69]^ | 2009 | Japan | Asian | 117/108 | PCR | CP | -863,-857 |  |  | YES |  | YES |
| Kobayashi ^[70]^ | 2009 | Japan | Asian | 147/303 | PCR | CP | -863 |  |  |  |  |  |
|  |  |  |  | 172/303 |  | AgP | -863 |  |  |  |  |  |
|  |  |  |  | 147/303 |  | CP | -857 |  |  |  |  |  |
|  |  |  |  | 172/303 |  | AgP | -857 |  |  |  |  |  |
| Moreira ^[49]^ | 2009 | Brazil | Mixed | 67/43 | PCR-RFLP | CP | -308 | YES |  |  |  |  |
|  |  |  |  | 55/43 |  | AgP |  | YES |  |  |  |  |
| Li ^[72]^ | 2009 | China | Asian | 100/118 | PCR-LDR | CP | -863 |  |  | YES |  |  |
| Sun ^[61]^ | 2009 | China | Asian | 107/138 | PCR-RFLP | CP | -308,-238 | YES | NO |  |  |  |
| Trombone ^[63]^ | 2009 | Brazil | Mixed | 127/177 | PCR-RFLP | CP | -308 | NO |  |  |  |  |
| Costa ^[27]^ | 2010 | Brazil | Mixed | 38/27 | PCR | CP | -308 | YES |  |  |  |  |
| Erciyas ^[36]^ | 2010 | Turkey | Caucasian | 35/85 | PCR-SSP | AgP | -308 | YES |  |  |  |  |
| Ricci ^[54]^ | 2011 | Italy | Caucasian | 32/23 | Real-time PCR | CP | -308 | YES |  |  |  |  |
| Liu ^[46]^ | 2011 | China | Asian | 99/50 | PCR-RFLP | CP | -308 | YES |  |  |  |  |
| Zhang ^[73]^ | 2011 | China | Asian | 115/115 | PCR-RFLP | CP | -863 |  |  | YES |  |  |
| Ma^[71]^ | 2011 | China | Asian | 96/104 | PCR-RFLP | CP | -308 | NO |  |  |  |  |
| Scapoli ^[26]^ | 2011 | Italy | Caucasian | 122/246 | PCR | AgP | -308 |  |  |  |  |  |
| Loo ^[68]^ | 2012 | China | Asian | 440/850 | PCR-RFLP | CP | -238 |  | NO |  |  |  |
| Garlet ^[41]^ | 2012 | Brazil | Mixed | 197/217 | Real-time PCR | CP | -308 | NO |  |  |  |  |
| Ianni ^[45]^ | 2013 | Italy | Caucasian | 75/440 | Real-time PCR | CP | -308 | YES |  |  |  |  |
| Yang ^[65]^ | 2013 | China | Asian | 180/180 | PCR-RFLP | CP | -308,-238,-1031,-857 | YES | YES |  | YES | YES |
|  |  |  |  | 180/180 |  | AgP |  | YES | YES |  | YES | YES |
| Ebadian ^[34]^ | 2013 | Iran | Asian | 58/60 | PCR-RFLP | AgP | -308 | YES |  |  |  |  |
| Sharma ^[59]^ | 2014 | India | Asian | 51/51 | PCR | CP | -308 | YES |  |  |  |  |
| Schulz ^[57]^ | 2014 | Germany | Caucasian | 53/89 | PCR-SSP | CP | -308,-238 | YES | YES |  |  |  |
| Özer Yücel ^[51]^ | 2015 | Turkey | Caucasian | 29/26 | PCR-RFLP | CP | -308 | NO |  |  |  |  |
|  |  |  |  | 38/26 |  | AgP |  | NO |  |  |  |  |
| Dosseva-panova ^[33]^ | 2015 | Bulgarian | Caucasian | 30/10 | PCR | CP | -308 | YES |  |  |  |  |
| Ho ^[44]^ | 2015 | China | Asian | 107/161 | PCR | CP | -308 | YES |  |  |  |  |
|  |  |  |  | 90/161 |  | AgP |  | YES |  |  |  |  |
| Barnea ^[19]^ | 2015 | Roman | Caucasian | 22/10 | Real-time PCR | AgP | -857 |  |  |  |  | YES |
| Grigorovich ^[42]^ | 2015 | Russia | Caucasian | 150/150 | PCR | CP | -308 | YES |  |  |  |  |
| Lavu ^[71]^ | 2016 | India | Asian | 169/171 | PCR | CP | -863 |  |  | NO |  |  |
|  |  |  |  | 145/163 |  | CP | -1031 |  |  |  | YES |  |
| Domínguez-Pérez ^[31]^ | 2017 | Mexico | Mixed | 80/80 | Real-time PCR | CP | -308 | YES |  |  |  |  |
| Majumder ^[20]^ | 2018 | India | Asian | 157/200 | PCR | CP | -308,-238,-863,-1031,-857 | NO | NO | NO | NO | YES |
|  |  |  |  | 40/200 |  | AgP |  | NO | NO | NO | NO | YES |

PCR: Polymerase Chain Reaction; PCR-RFLP: restriction fragment length polymorphism of polymerase chain reaction; PCR-SSP: polymerase chain reaction with sequence-specific primers; HWE: Hardy-Weinberg equilibrium.
